# Contemporary and historical evolutionary processes interact to shape patterns of within-lake phenotypic divergences in polyphenic pumpkinseed sunfish, *Lepomis gibbosus*

**DOI:** 10.1002/ece3.72

**Published:** 2012-03

**Authors:** Dylan J Weese, Moira M Ferguson, Beren W Robinson

**Affiliations:** Department of Integrative Biology, University of Guelph,Guelph, Ontario N1G 2W1, Canada

**Keywords:** Trophic polymorphism, resource polymorphism, trophic morphology, glacial refugia, phylogeography, divergent natural selection, ecomorphology, geometric morphometrics, Centrarchidae, postglacial lakes, *Lepomis gibbosus*, Centrarchid sunfish

## Abstract

Historical and contemporary evolutionary processes can both contribute to patterns of phenotypic variation among populations of a species. Recent studies are revealing how interactions between historical and contemporary processes better explain observed patterns of phenotypic divergence than either process alone. Here, we investigate the roles of evolutionary history and adaptation to current environmental conditions in structuring phenotypic variation among polyphenic populations of sunfish inhabiting 12 postglacial lakes in eastern North America. The pumpkinseed sunfish polyphenism includes sympatric ecomorphs specialized for littoral or pelagic lake habitats. First, we use population genetic methods to test the evolutionary independence of within-lake phenotypic divergences of ecomorphs and to describe patterns of genetic structure among lake populations that clustered into three geographical groupings. We then used multivariate analysis of covariance (MANCOVA) to partition body shape variation (quantified with geometric morphometrics) among the effects of evolutionary history (reflecting phenotypic variation among genetic clusters), the shared phenotypic response of all populations to alternate habitats within lakes (reflecting adaptation to contemporary conditions), and unique phenotypic responses to habitats within lakes nested within genetic clusters. All effects had a significant influence on body form, but the effects of history and the interaction between history and contemporary habitat were larger than contemporary processes in structuring phenotypic variation. This highlights how divergence can be better understood against a known backdrop of evolutionary history.

## Introduction

Trait differences among populations and species are often interpreted as being the result of contemporary evolutionary processes, such as adaptation to current ecological conditions, or the result of historical evolutionary process such as bottle necks, genetic drift, or adaptation to past ecological conditions. Comparative approaches are frequently used to test for adaptation to ecological conditions by demonstrating a significant correlation between current conditions and organismal traits in an attempt to reject random chance as an explanation for trait differences among populations (Robinson et al. 2000; Jastrebski and Robinson 2004; Millar et al. 2006). Unexplained variation in these methods is often attributed to the vagaries of historical evolutionary events or unmeasured ecological variables. The role of historical evolutionary process on contemporary trait variation can be tested by evaluating the correlation between variation at neutral genetic markers and traits, thereby supporting the notion that historical events that influenced neutral markers also influenced trait evolution (Merilä 1997; Merilä and Crnokrak 2001; Hankison and Ptacek 2008). A failure to detect such an historical correlation might be attributed to selection without necessarily understanding the ecological source of selection.

More likely though, is that both contemporary and historical processes contribute to trait variation among species and populations (Taylor and McPhail 2000; Berner et al. 2010; Rosenblum and Harmon 2011). Furthermore, as Langerhans and DeWitt (2004) point out, responses to contemporary selection may be influenced by historical evolutionary responses because these shape the nature of genetic variation and covariation within contemporary populations. Thus, selection imposed by similar contemporary ecological conditions may result in different evolutionary responses depending on evolutionary history. Under this hypothesis, contemporary adaptive phenotypic evolution is dynamically influenced by evolutionary history.

Disentangling the effects of historical from contemporary processes and the dynamic effects of history interacting with contemporary processes is possible when a phylogeny accurately describes the evolutionary relationships among species; or within species, population genetics is used to establish patterns of genetic structure among populations. Significant historical processes are expected to cause phenotypic similarities among populations nested in lineages that reflect patterns of common ancestry (Felsenstein 1985; Harvey and Pagel 1991; Langerhans and DeWitt 2004; Riopel et al. 2008). A significant correlation between population traits and environmental features supports the role of selection imposed by contemporary ecological conditions; however, in the absence of phylogenetic context, such a pattern might be variously consistent with historical or contemporary processes. On the one hand, a dominant role for evolutionary history is supported when phenotypically divergent ecomorphs occupy similar habitats within a species and ecomorphs are descended from different ancestral lineages (Bernatchez 1997). In this case, there is limited evidence for the role of adaptation to contemporary ecological conditions since the association between habitat and phenotype is unreplicated, and adaptive arguments must be based solely on logic derived from biophysical first principles. On the other hand, the parallel evolution of similar traits in similar environments among populations representing independent evolutionary lineages suggests a dominant role for adaptation to contemporary conditions (Taylor and Bentzen 1993; Bernatchez et al. 1996; Pigeon et al. 1997; Gislason et al. 1999; Taylor and McPhail 1999; Schluter et al. 2004; Ostbye et al. 2006). Interactions between historical and contemporary processes can be detected by statistical models that explicitly evaluate if trait variation among populations is affected by ancestry (using population genetics), contemporary environmental conditions, and interactions between ancestry and environment (Langerhans and DeWitt 2004).

Studies investigating the phylogeography (Wilson and Hebert 1996; Bernatchez and Wilson 1998; Poissant et al. 2005; Aldenhoven et al. 2010), ecology (Mandrak and Crossman 1992; Robinson et al. 2000; McCairs and Fox 2004; Parsons and Robinson 2007), and phenotypic evolution (Pigeon et al. 1997; Gislason et al. 1999; Jastrebski and Robinson 2004) of fish inhabiting postglacial lakes in the northern hemisphere have provided many valuable insights regarding how historical and contemporary processes interact to influence patterns of genetic and phenotypic variation among populations. However, few studies have explicitly combined population genetic and phenotypic variables in a single analysis that permits a quantitative assessment of the relative importance of historical and contemporary factors on phenotypic variation among diverging populations (Ward and Mclennan 2009; Berner et al. 2010). Here, we evaluate how contemporary and historical evolutionary processes interact to influence the intraspecific phenotypic diversity of pumpkinseed sunfish (Lepomis gibbosus) in a postglacial landscape.

### Pumpkinseed sunfish polyphenism

The pumpkinseed sunfish of North America exemplifies how a variety of mechanisms contribute to phenotypic diversity. Pumpkinseed sunfish are a common species with a wide distribution in northeastern North America that is more northerly than all other sunfish, including a substantial region of postglacial environment (Scott and Crossman 1998). Its functional morphology includes specialized traits for feeding on large armored invertebrates such as snails (Lauder 1983; Wainwright et al. 1991), which likely provides a competitive refuge from other sunfish (Mittelbach 1984). In the absence of bluegill sunfish (L. macrochirrus), a zooplankton generalist, some pumpkinseed sunfish are found in open water (pelagic) habitats of postglacial lakes, where they feed on zooplankton and are phenotypically divergent from local pumpkinseed sunfish that inhabit the shallow inshore littoral habitat (Robinson et al. 1993; Robinson et al. 2000; Gillespie and Fox 2003; Jastrebski and Robinson 2004). Phenotypic diversity within numerous lakes in New York State (USA) and the province of Ontario (Canada) is thus represented by a trophically related polyphenism bounded by sympatric littoral and pelagic ecomorphs (Robinson et al. 1993; Robinson et al. 2000; Gillespie and Fox 2003; Jastrebski and Robinson 2004).

We have been exploring the mechanisms that shape phenotypic diversity in pumpkinseed sunfish that inhabit postglacial lakes. Despite physical and ecological differences among lakes in this study system (Robinson et al. 2000), consistent associations between morphology (body shape and gill-raker length) and habitat (Robinson et al. 2000; Gillespie and Fox 2003; Jastrebski and Robinson 2004) and trade-offs between foraging ability and phenotype (Robinson et al. 1996) both suggest that within-lake patterns of diversity are influenced by contemporary patterns of diversifying selection between littoral and pelagic habitats that are qualitatively similar among lakes. The influence of historical evolutionary processes on shaping the pumpkinseed polyphenism though, has not been formally considered.

Historical evolutionary processes may also shape phenotypic variation here because polyphenism differs between related species of bluegill and pumpkinseed sunfish (Riopel et al. 2008) and by meristic variation among pumpkinseed sunfish over a relatively large geographic scale (western populations have higher numbers of anal and dorsal fin rays than eastern populations; Scott and Crossman 1998). This pattern is potentially explained by the historical isolation of ancestral populations in multiple glacial refugia (Pielou 1991). Pumpkinseed sunfish were likely present in both the Mississippian and the Atlantic refugia during the Wisconsian glaciation event (Underhill 1986; Mandrak and Crossman 1992) and both central Ontario and New York state could have been colonized by ancestral populations from either, or both, of the Atlantic or Misissippian refugia (Smith 1985; Underhill 1986; Mandrak and Crossman 1992). Thus, the allopatric divergence of ancestral pumpkinseed populations that subsequently colonized postglacial aquatic habitats throughout this region over the last 15,000 years could account for some of the within lake diversity. Despite an appreciation of diverse ultimate and proximate mechanisms shaping phenotypic diversity in pumpkinseed sunfish, we know little about the potential role of any historical processes in shaping phenotypic variation among populations. If ancestral pumpkinseed diverged and contemporary lake populations were colonized by different or multiple ancestral sources, then some of the phenotypic diversity currently expressed among- and/or within-lake populations may reflect these historical, in addition to contemporary, evolutionary processes. The purpose of this study is to test for such historical effects using a combination of population genetic and morphological methods. Specifically, we evaluate two hypotheses about the role of historical effects.

1. An extreme possibility is that the phenotypic divergence between littoral and pelagic ecomorphs occurred only once in allopatry (potentially between glacial refugia), and that descendants of these populations dispersed and subsequently colonized lakes resulting in contemporary populations of polyphenic pumpkinseed sunfish. This single historical origin hypothesis predicts that pumpkinseeds sampled from littoral and pelagic habitats from the same lake should be distantly related, and that pumpkinseeds sampled from the same habitat type from different lakes should be closely related. We evaluate these predictions using allelic variation at six microsatellite loci.
2. A less extreme possibility is that ecomorphs within lakes have multiple contemporary origins, but that more recent historical effects nonetheless influence broad-scale patterns of phenotypic variation across the postglacial geographical landscape. This multiple historical origin hypothesis predicts that populations of pumpkinseed with different evolutionary histories have different phenotypes, regardless of contemporary mechanisms that currently structure diversity within lakes. Additionally, historical effects and adaptation to contemporary habitats do not preclude each other, and might interact (Langerhans and DeWitt 2004). We use variation in allelic frequencies at six microsatellite loci to group populations into genetic clusters with shared evolutionary histories. We then test for the effects of contemporary selection (similarities in within-lake phenotypic divergence among clusters), history (phenotypic differences between genetic clusters), and interactions between history and selection (unique aspects of within-lake divergence among clusters) on phenotypic diversity (Langerhans and DeWitt 2004).

## Methods

### Field collections

**Figure 1:**
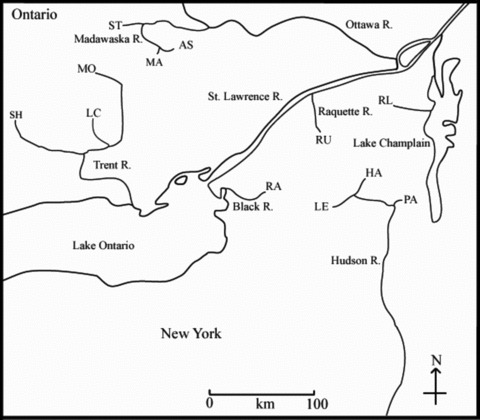
Schematic illustration of locations and contemporary drainage connections among the 12 lakes used in this study. In Ontario, Ashby (AS), Mayo (MA), and Salmon Trout (ST) drain into the Ottawa River through the Madawaska River (Ottawa R. drainage). Looncall (LC), Shadow (SH), and Monck (MO) drain into the north side of Lake Ontario through the Trent River (Trent R. drainage). In the Adirondack region of northern New York State, three lakes drain north into the south-shore St. Lawrence River drainage. Lake Rondaxe (RA) drains through the Black river into the southeast side of Lake Ontario; Round Lake (RU) and Rollins Pond (RL) drain into the St. Lawrence River through the Raquette River and Lake Champlain, respectively. Lakes that drain south through the Adirondack region empty through the Hudson River drainage to the Atlantic Ocean and include Harris (HA), Paradox (PA), and Lewey (LE).

Pumpkinseed sunfish were collected between May and September 2002 from 12 lakes (hereafter referred to as “lake populations”), in which pumpkinseeds inhabit both the littoral and pelagic habitats (Table 1; Fig. 1). We created a balanced sampling design by choosing three lakes within each of four contemporary drainage systems, two drainages in each of two regions (Ontario and New York, approximately midway between glacial refugia in the Mississippi drainage, and along the Atlantic seaboard). Approximately 50 individuals from each littoral and pelagic habitat in every lake were collected using sampling techniques consistent with previous studies (Robinson et al. 2000; Gillespie and Fox 2003; Jastrebski and Robinson 2004). Fish were collected by angling, or captured in fish traps (35-cm diameter × 90-cm long; 8-cm diameter aperture). Pelagic sunfish were designated as those collected from habitat adjacent to deepwater, such as islands and shoals in the main lake basin, and from points that extend into the main lake basin (>2 m depth). Littoral sunfish were designated as those sampled from shallow water (<2 m depth) close to shore, and at the back of bays, both usually characterized by extensive macrophyte growth. A study of individual movement patterns revealed that both ecomorphs exhibit strong habitat fidelity supporting the ecological distinctness of ecomorph populations (McCairns and Fox 2004). The sample of fish collected from a particular habitat in a lake represents the ecomorph population. Thus, two ecomorph populations were sampled in each lake, for a total of 24 ecomorph populations. A section of a pectoral fin from every individual was removed and stored in 95% ethanol for genetic analysis. Approximately the first 20 fish collected from each habitat in each lake were euthanized using 250 ppm clove oil, preserved in 10% buffered formalin for 3 months then rinsed with water, and stored in 70% ethanol. The whole specimens were subsequently used for morphological analysis (see next).

**Table 1:** Locality information and sample sizes for the pelagic and littoral subpopulations inhabiting the 12 study lakes. Numbers in parentheses beside sample sizes (*n*) are the numbers of individuals in the subsample used for phenotypic analyses (see Methods).

Lake (Abbv.)	Longitude	Latitude	Region	Drainage	*n* pelagic	*n* littoral
Ashby (AS)	45°05′N	77°21′W	Ontario	Ottawa	48(19)	48(21)
Mayo (MA)	45°02′N	77°35′W	Ontario	Ottawa	48(20)	48(18)
Salmon Trout (ST)	45°11′N	77°49′W	Ontario	Ottawa	48(20)	48(23)
Looncall (LC)	44°45′N	78°11′W	Ontario	Lake Ontario	48(19)	48(21)
Monck (MO)	45°00′N	78°07′W	Ontario	Lake Ontario	48(20)	34(20)
Shadow (SH)	44°43′N	78°48′W	Ontario	Lake Ontario	48(20)	48(20)
Rollins (RL)	44°38′N	74°23′W	New York	St. Lawrence	47(20)	47(21)
Round (RU)	44°05′N	74°34′W	New York	St. Lawrence	48(21)	47(20)
Rondaxe (RA)	43°45′N	74°54′W	New York	St. Lawrence	46(20)	48(22)
Paradox (PA)	43°30′N	73°41′W	New York	Hudson	48(20)	48(20)
Harris (HA)	43°58′N	74°07′W	New York	Hudson	45(22)	48(20)
Lewey (LE)	43°38′N	74°23′W	New York	Hudson	48(20)	48(21)

### Microsatellite genotyping

DNA was isolated from pectoral fin tissue using a phenol/chloroform/isoamyl alcohol extraction method (Bardacki and Skibinski 1994). Six loci with reproducible and clear amplification profiles (Table 2) were selected and amplified using the polymerase chain reaction (PCR) in 10.7 µl reactions (5 µl DNA [6 ng/µl)] with 5.7µl PCR cocktail). The PCR cocktail consisted of 10× PCR buffer (20 mM Tris-HCl, 50 mM KCL), 1.5 mM MgCl_2_, 0.136 µM dNTP, 0.36 µM dCTP, 0.45 µM of forward and reverse primers, 0.09 mg/mL BSA, 0.25 units of Taq DNA polymerase. Three loci (Lma 29, RB20, and RB7) were amplified under the following temperature regime: 95°C for 5 min; 35 cycles of 95°C for 30 sec, annealing temperature (T_A_) for 1 min, 72°C for 1 min; then another 95°C for 30 sec, T_A_ for 1 min, and finally 72°C for 10 min. The remaining loci (Lmar 9, Lmar14, and Lmar18) were amplified using a shorter program: 95°C for 5 min; 35 cycles of 95°C for 30 sec, T_A_ for 45 sec, 72°C for 1 min. The final cycle was followed by 72°C for 5 min. The PCR products were separated on 6% acrylamide gels in TBE buffer and then visualized with a Hitachi FMBIO fluorescent imaging system (Hitachi, San Francisco, CA, USA) Allele sizes were determined, using FMBIO software, by comparing the fragment size from the samples to several known lane standards (350 or 500 TAMRA depending on allele size) included with every gel.

**Table 2:** Six microsatellite loci used to study population genetic structure of pumpkinseed sunfish in central Ontario and the Adirondack region of New York State. Included are repeat motif, annealing temperature (T_A_), source species in genus Lepomis from which these loci were isolated, and original reference.

Microsatellite	Repeat motif	T_A_ (°C)	Source species	Reference
Lma 29	(GT)_n_	58	*L. macrochirus*	Colbourne et al. (1996)
RB7	(GATA)_n_	58	*L. auritus*	DeWoody et al. (1998)
RB20	(GATA)_n_	58	*L. auritus*	DeWoody et al. (1998)
Lmar9	(AGAT)_n_	60	*L. marginatus*	Schable et al. (2002)
Lmar14	(AGAT)_n_	62	*L. marginatus*	Schable et al. (2002)
Lmar18	(AGAT)_n_	58	*L. marginatus*	Schable et al. (2002)

### Microsatellite polymorphism and within-lake genetic differentiation

MSA software (Dieringer and Schlotterer 2002) was used to calculate allele frequencies, number of alleles, and observed and expected heterozygosity for each locus in every ecomorph population. Deviation from Hardy–Weinberg proportions within each ecomorph population was tested by the exact test for each locus and population using GENEPOP version 3.3 (Raymond and Rousset 1995). The same program was used to test for linkage disequilibrium between loci within ecomorph populations, and to implement Fisher's exact test of allelic frequency homogeneity between every combination of ecomorph populations. To specifically determine if sympatric ecomorph populations are genetically heterogeneous, F_st_ values were calculated between ecomorphs in each lake using FSTAT (Goudet 1995). Population differentiation was considered significant if the confidence interval for the multilocus estimate of F_st_, resulting from 1000 data permutations, excluded zero. To compare allelic richness between fish from different regions and drainages, *t*-tests (with Bonferroni adjusted P-values) were used to compare the number of alleles in each population, averaged over all six loci.

### Population genetic structure

There was very limited evidence of genetic divergence between ecomorphs within lakes (see Results); therefore, ecomorph populations from particular lakes were pooled for all following analyses of geographic patterns of genetic structure. Patterns of genetic structuring were assessed using both *F*-statistics, which do not incorporate differences in allele size, and *R*-statistics, which incorporate the number of repeated units in a microsatellite as information (assuming the stepwise model of microsatellite mutation). We used Arlequin (Schneider et al. 2000) to calculate pairwise values of F_st_ and R_st_ between all populations, significance was assessed with 1000 permutations. The allele size permutation test was implemented in the program SPAGeDi 1.1 (Hardy and Vekemans 2002). This procedure performs 2000 permutations of a given dataset with randomized allele sizes, and then compares observed R_st_ values to the distribution of permutated values. *R*-statistics are appropriate when observed Global R_st_ is improbably large (P < 0.05) compared to the permutated R_st_ values; *F*-statistics are appropriate when the observed R_st_ value is similar to the permutated R_st_ values.

We tested the effect of contemporary drainage distances on geographical patterns of among lake population genetic structure. First, to partition allelic variation among different geographic effects, we performed an analysis of molecular variance (AMOVA) implemented in ARLEQUIN using both F_st_ and R_st_ values. For this AMOVA, lake populations (ecomorphs pooled) were nested within watersheds. Second, we tested for isolation by distance (IBD) by evaluating the relationship between pairwise genetic distance and geographical distance between populations. Google Earth was used to calculate fluvial distance among all lakes in the St. Lawrence, Ottawa, and Lake Ontario drainages. The lake populations from the Hudson River drainage were not included in the IBD analyses, because contemporary gene flow between the Hudson populations and the rest of the study lakes along contemporary connections would require the movement of pumpkinseed through the marine environment (pumpkinseed are not known to inhabit saltwater). Pairwise F_st_ and R_st_ values (see above) were transformed to F_st_/1-F_st_ and R_st_/1-R_st_ (Rousset 1997) and the program IBDWS (Jensen et al. 2005) was used to perform separate Mantel tests for each distance measure (10,000 permutations) to determine if there was a significant IBD relationship.

Population clusters were assessed using two separate approaches. (1) We used Cavalli–Sforza and Edward's (1967) chord distance (D_ce_) to construct a consensus neighbor-joining phenogram among lake populations using software contained in the PHYLIP 3.5 package (Felsenstein 1993). Statistical support for nodes was obtained from 1000 bootstrapped replicates of the original dataset. When reconstructing historical population relationships between recently diverged populations, studies have shown that D_ce_, which does not assume equal population sizes or mutation rates between loci, is more likely to achieve the correct tree topology compared to other methods (Takezaki and Nei 1996; Goldstein and Pollock 1997). Thus, we use D_ce_ because we are chiefly interested identifying the most accurate branching topology for our phenogram, so that groups of populations sharing a recent common ancestor can be identified for morphological analyses (see next). (2) We also used the program STRUCTURE 2.1 (Pritchard et al. 2000) to infer the number of genetic clusters independently of geographic sampling using a posteriori Bayesian genotype clustering. We used the admixture model with a burn-in period of 10,000 and 10,000 iterations and tested models with values of *K* (number of clusters) ranging from 1 to 20. The correction of Evanno et al. (2005) was implemented to determine the most probable value of *K* from the posterior probabilities for each *K*. We also assessed contemporary geographical population structure by visualizing the results of STRUCTURE models with constrained levels of *K*= 2 and *K*= 3 (Garnier et al. 2004; Aldenhoven et al. 2010).

### Influence of evolutionary history on phenotypic variation and divergence

The results of the above analyses of population genetic structure informed further tests of the effect of evolutionary history on contemporary within-lake divergence of pumpkinseed ecomorphs. Specifically, we implemented the statistical tests suggested by Langerhans and DeWitt (2004), which involve quantifying shared and unique responses of different evolutionary lineages to a similar environmental gradient (Langerhans et al. 2003, 2006; Foster et al. 2008; Langerhans and Makowicz 2009; Ward and Mclennan 2009; Ruehl et al. 2011). In our case, the results of our population genetic analyses persistently suggest that Ontario populations of pumpkinseed are distinct from New York lake populations, which are further subdivided by drainage—Hudson versus St. Lawrence (see Results). These particular genetic clusters were useful because they each contained replicate lakes (at least three lakes within each clusters) that allowed us to statistically evaluate the effect of history on phenotypic divergence by considering divergence in multiple lakes. Earlier work in this study system has documented parallel (shared) divergence of littoral and pelagic ecomorphs among lake populations of pumpkinseed (Robinson et al. 2000; Jastrebski and Robinson 2004). The objective of the current analysis is to determine if the diversity in evolutionary histories (among Ontario, St. Lawrence, and Hudson genetic clusters), documented in this study, is associated with contemporary phenotypic variation; and, to test for unique aspects of divergence among lake populations nested within the different genetic clusters.

**Figure 2:**
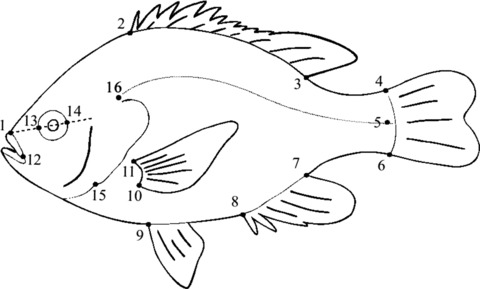
Illustration of the 16 landmarks placed on the left side of each pumpkinseed sunfish, L. gibbosus, collected from 12 lakes in central Ontario and the Adirondack region of New York state, for use in the morphometric analysis of external body shape.

Variation in body shape was assessed using geometric morphometrics. Photographs of each fish were taken with a Nikon 950 digital camera (Nikon Corp., Tokyo, Japan). TPSDIG (Rohlf 2003) was used to locate the *X* and *Y* coordinates of 16 homologous landmarks on the left side of each fish (Fig. 2). TPSRELW (Rohlf 2003) was used to rotate, translate, and scale landmark coordinates through generalized least squares superimposition. From these superimposed landmark coordinates, TPSRelw also calculates affine (two uniform components) and nonaffine (26 partial warps) components of shape variation (Parsons et al. 2003).

Nested MANCOVA models, with all partial warps and uniform components as dependent variables, were used to test for shared and unique features of divergence (Langerhans et al. 2003, 2006; Langerhans and DeWitt 2004; Hendry et al. 2006; Ward and Mclennan 2009). Throughout, we tested for allometry using centroid size as a covariate. First, we implemented a model that considered all 12 lakes from the three genetic clusters (Ontario, St. Lawrence, and Hudson). We tested for shared aspects of divergence (between littoral and pelagic ecomorphs) among lake populations using ecomorph (pelagic or littoral) as a fixed factor. We also tested for the influence of evolutionary history on phenotypic variation using genetic cluster (Ontario, St. Lawrence, or Hudson) as a fixed factor. The influence of evolutionary history on divergence (unique aspects of divergence) was tested by including an interaction term between ecomorph and cluster. We tested for the influence of lake on phenotypic variation using lake nested within genetic cluster as a factor. The relative influence of each factor in the model was estimated using Wilks’ partial variance statistic (η^2^). Canonical variates associated with each effect of interest were retained and used to produce thin-plate spline diagrams using TPSREGR (Rohlf 2003) to visualize morphological variation. Note that shape deformations along these canonical axes do not represent the original shape space and so are cautiously interpreted (Foster et al. 2008). The above model detected a significant effect of evolutionary history on phenotypic divergence (genetic cluster × ecomorph interaction—see Results) and so we performed additional MANCOVA models intended to investigate patterns of divergence within genetic clusters (considering lakes within each of the three clusters separately). Specifically, using the uniform components and partial warps of morphological variation as dependent variables, we tested for the effects of allometry (using centroid size as a covariate), shared aspects of divergence (parallel diversity) among lakes within a cluster (ecomorph as a factor), the influence of evolutionary history on phenotypic variation (lake as a factor), and the influence of evolutionary history on divergence (interaction between ecomorph and lake as a factor).

## Results

### Microsatellite polymorphism and within-lake genetic differentiation

The six microsatellite loci all showed moderate to high levels of polymorphism (Table S1). In many populations, H_e_ was above 0.90 for certain loci. Observed heterozygosity was consistently lower than expected in the Ontario ecomorph populations for Lmar14 (10/12 populations). All other loci exhibited approximately the number of deviations from HWE than would be predicted by chance (at α= 0.05) (5/120 populations). There was no significant linkage disequilibrium detected between pairs of loci in any ecomorph population. *T*-tests between the mean numbers of alleles per population averaged over all six loci indicated that the six New York populations had more alleles than the six Ontario populations (P < 0.005). No significant differences in the number of alleles were found between drainages after adjustment of P-values following the Bonferoni correction. A number of private alleles shared between local ecomorph pairs (the littoral and pelagic ecomorphs found in the same lake) were also discovered in five Adirondack lakes: Lma29 allele 105 is only found in the Harris lake population; RB20 allele 303 is only found in the Lake Rondaxe population; Lmar18 alleles 283 and 287 are only found in Paradox lake; RB7 allele 314 is only found in Round lake; Lmar14 allele 411 is only found in Lewey lake.

The null hypothesis of homogeneity of allele frequencies was rejected, using Fisher's exact test, in all pair-wise comparisons of ecomorph populations (P < 0.001), excluding comparisons between sympatric ecomorphs within lakes. There was only very limited evidence of genetic differentiation between pelagic and littoral ecomorphs within lake populations. Between local ecomorphs, the null hypothesis of homogeneity of allele frequencies was rejected (P < 0.05) in two lakes: Round Lake in the St. Lawrence drainage and Lewey Lake in the Hudson drainage. However, multilocus pairwise F_st_ values between local ecomorphs were very low (<0.008), and only in one lake (Round Lake) was it significantly different from zero (F_st_= 0.005). Thus, ecomorph populations from the same lake were pooled in order to investigate broader geographic patterns of genetic structure.

### Population genetic structure

Allele size permutation tests revealed that geographical variation in allele sizes contributed useful phylogeographic information (P < 0.05 in all tests); therefore, we present the results of both F_st_- and R_st_-based distances. All multilocus, pairwise estimates of F_st_ and R_st_ were greater than zero (P < 0.01) (Table 3). The hierarchical (AMOVA) analyses of genetic variance revealed that allelic variation is attributable to geographical entities (Table 4). We detected significant patterns of population structure reflecting lake and drainage membership. We note that in all AMOVA tests of genetic structure between geographical entities (as opposed to within populations), calculations of R_st_ explained more variation than calculations of F_st_. When only F_st_ calculations were considered, allelic differences among drainages explained less variation than allelic variation among lakes within drainages (3.9% and 7.6%, respectively); however, when only R_st_ values were considered, variation among drainages explained a greater proportion of molecular variation than variation among lakes within drainages (27.2% and 13.7%, respectively). Despite the AMOVA results (which detected significant effects of geographical entities), mantel tests comparing genetic (R_st_/1-R_st_ and F_st_/1-F_st_) and geographic distance matrices found no significant pattern of IBD among these lake populations (St. Lawrence, Ottawa, and Lake Ontario drainages: all *P* > 0.1). Taken together, pumpkinseeds sampled from particular lakes and drainages seem to be closely related to each other, however, these affiliations do not seem to reflect distances among contemporary waterways (see next).

**Table 3:** Estimates of multilocus pairwise R_st_ (above diagonal) and F_st_ (below diagonal) values based on variation at six polymorphic microsatellite loci among the 12 lake populations of pumpkinseed sunfish (lake abbreviations found in Table 1). All values were significantly greater than zero (*P* < 0.01).

	AS	MA	ST	MO	LC	SH	RL	RU	RA	PA	LE	HA
AS	–	0.120	0.159	0.094	0.098	0.050	0.371	0.792	0.405	0.112	0.177	0.254
MA	0.095	–	0.293	0.123	0.220	0.144	0.333	0.778	0.369	0.121	0.281	0.223
ST	0.104	0.137	–	0.147	0.111	0.114	0.290	0.782	0.311	0.125	0.391	0.158
MO	0.160	0.180	0.095	–	0.077	0.044	0.294	0.781	0.325	0.050	0.288	0.167
LC	0.099	0.109	0.097	0.144	–	0.029	0.279	0.765	0.319	0.038	0.227	0.156
SH	0.040	0.060	0.091	0.135	0.061	–	0.296	0.766	0.328	0.040	0.207	0.170
RL	0.138	0.109	0.157	0.183	0.102	0.093	–	0.228	0.016	0.242	0.457	0.042
RU	0.153	0.138	0.172	0.193	0.113	0.110	0.025	–	0.274	0.684	0.797	0.445
RA	0.177	0.176	0.198	0.217	0.128	0.150	0.048	0.033	–	0.250	0.490	0.069
PA	0.133	0.129	0.134	0.170	0.068	0.085	0.061	0.066	0.080	–	0.192	0.133
LE	0.131	0.129	0.149	0.180	0.114	0.094	0.072	0.078	0.111	0.083	–	0.382
HA	0.068	0.075	0.077	0.114	0.056	0.039	0.048	0.042	0.074	0.050	0.060	–

**Table 4:** Hierarchical AMOVA based on six microsatellite loci in populations of pumpkinseed sunfish from lakes in central Ontario and the Adirondack region of New York State. AMOVA was used to test the hypothesis that contemporary drainage structure explains patterns of genetic variation by pooling ecomorphs within lakes and nesting lakes within drainages. For each parameter, upper values are based on allele frequency only (F_st_) and lower values considered variation in allele size and allele frequency (R_st_). Listed are variance (V), proportion of variance explained by a particular grouping (%), fixation index (haplotypic correlation at corresponding grouping), and the probability of having a more extreme variance component than that observed (P).

Effect	V	%	Fixation indices	P-value
Among drainages	0.093	3.93	0.039	<0.001
	40.7	27.2	0.037	<0.001
Among lakes within	0.18	7.61	0.079	<0.001
drainages	20.5	13.7	0.081	<0.001
Among individuals	2.1	88.4	0.115	<0.001
within lakes	88.5	59.1	0.115	<0.001

**Figure 3:**
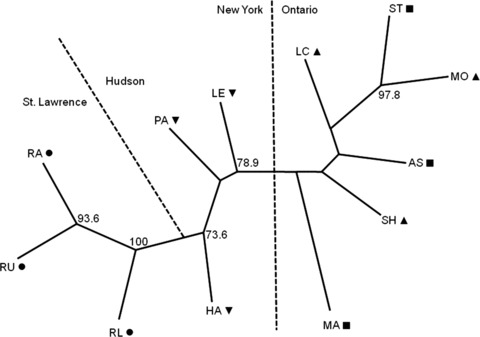
Phenogram depicting relationships among the 12 lake populations of pumpkinseed sunfish sampled from Central Ontario and the Adirondack region of New York State. Superscripts correspond to drainage systems: Ottawa R. drainage of Ontario (▪); Trent R. drainage of Ontario (▴); St. Lawrence R. drainage of New York (•); Hudson R. drainage of New York (▾). Relationships were inferred from a matrix of D_ce_ genetic distances and the Neighbor-joining algorithm. For 1000 bootstrap replicates, node values of 50% and higher are shown. This analysis grouped population from Ontario together with 78.9% bootstrap support, populations from the St. Lawrence drainage of New York with 100% bootstrap support.

The neighbor-joining phenogram based on D_ce_ distances had a robust topology with several nodes being supported in over 70% of the bootstrap replicates (Fig. 3). All Ontario populations form a reasonably well-supported cluster separate from the New York populations (78.9% of bootstrap replicates). In New York, the populations corresponding to the St. Lawrence drainage form a well-supported (100% bootstrap support) grouping, separate from the Hudson River drainage populations—which were more closely related to Ontario populations. In contrast, populations within contemporary drainage systems in Ontario do not form monophyletic groupings; for example, populations from Monck and Salmon Trout lakes, which are from different Ontario drainages, form a monophyletic cluster with 97.8% bootstrap support.

**Figure 4:**
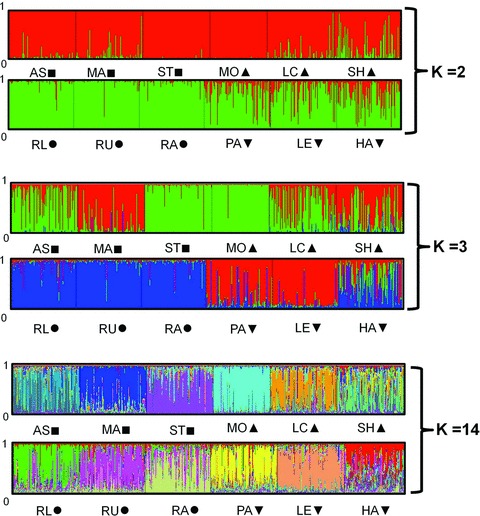
Bar plot of membership proportions for all individual from the 12 lakes (represented by abbreviations, see methods) in this study using STRUCTURE for *K*= 2, *K*= 3, and *K*= 14 (most likely). Symbols correspond to drainage systems: Ottawa R. drainage of Ontario (▪); Trent R. drainage of Ontario (▴); St. Lawrence R. drainage of New York (•); Hudson R. drainage of NewYork (▾).

Individual-based clustering using STRUCTURE generally identified population clusters associated with individuals sampled from the 12 different lakes. However, the most probable number of clusters, based on the approach of Evanno et al. (2005), in our dataset was actually *K*= 14 (Table 5), indicating more population genetic clusters than lakes. Under the *K*= 14 STRUCTURE analyses, two clusters (5 and 12) contain relatively high numbers of individuals from different lakes. Cluster 5 includes individual fish from Rollins, Round, and Rondaxe lakes from the Hudson River drainage, cluster 12 includes individual fish from Ashby and Shadow lakes from Ontario (Fig. 4; Table 6). We interpret these extra clusters to be an artifact of how recently these lakes have diverged from each other, and we are not aware of any recent introductions or transfers of pumpkinseeds among lakes that could produce this pattern. Furthermore, these lakes were not those with marginal support for allelic heterogeneity between ecomorphs, suggesting that these genetic clusters do not reflect differences in ecological specialization. Individuals from all other lakes in the *K*= 14 model were generally assigned to lake-specific clusters with relatively high frequency (Table 6). We also used STRUCTURE to assess whether the results of the Bayesian individual-based clustering approach generally corroborated the more basal geographical groupings suggested by the D_ce_ phenogram. Specifically, when we constrained STRUCTURE to estimate two clusters (*K*= 2), individuals from Ontario were generally distinguished from individuals from New York (Fig. 4), consistent with the D_ce_ phenogram. Also, when we constrained STRUCTURE to estimate three clusters (*K*= 3), individuals from New York were further subdivided into drainage-specific clusters (Fig. 4), consistent with the D_ce_ phenogram.

**Table 5:** Likelihood of various numbers of genetic clusters (*K*) based on the STRUCTURE 2.2 analysis of all 1129 individual pumpkinseed sunfish from the 12 lake populations considered in this study. Following Bayes’ rule the probability (PrK) for *K*= 14 was estimated to be 1.

K	ln Pr (*X* |K)	PrK
1	–31,640.7	0
2	–29,765	0
3	–28,887.1	0
4	–28,299.5	0
5	–27,777.9	0
6	–28,633.8	0
7	–27,254	0
8	–27,045.4	0
9	–26,963.2	0
10	–26,782.3	0
11	–26,719.2	0
12	–26,642	0
13	–26,790.5	0
14	–26,528.8	1
15	–26,764.5	0
16	–29,727.2	0
17	–26,866.9	0
18	–300,066.1	0
19	–27,138.1	0
20	–28,410.2	0

**Table 6:** Results from STRUCTURE based on the most likely number of cluster (*K*= 14). Proportion of genotypes from each sampling site within each of the 14 inferred clusters are shown. Bold values indicate proportions greater than 0.1.

Inferred cluster														
Lake	1	2	3	4	5	6	7	8	9	10	11	12	13	14
AS	0.017	0.009	0.041	0.014	0.007	0.019	0.031	0.02	0.018	**0.383**	0.089	**0.318**	0.025	0.01
MA	0.019	0.014	**0.718**	0.016	0.011	0.019	0.028	0.023	0.017	0.03	0.055	0.02	0.022	0.009
ST	0.018	0.008	0.018	0.014	0.006	0.067	0.043	0.016	0.01	0.035	0.023	0.052	**0.684**	0.007
MO	0.009	0.006	0.011	0.007	0.006	**0.837**	0.012	0.008	0.007	0.017	0.015	0.028	0.034	0.006
LC	0.028	0.012	0.034	0.021	0.012	0.035	**0.581**	0.049	0.018	0.063	0.053	0.041	0.041	0.012
SH	0.045	0.021	0.05	0.056	0.015	0.046	0.086	0.089	0.024	0.073	**0.33**	0.112	0.036	0.017
RL	0.038	**0.582**	0.018	0.024	**0.136**	0.013	0.01	0.056	0.018	0.014	0.019	0.016	0.013	0.041
RU	0.071	0.08	0.016	0.014	**0.545**	0.017	0.012	0.069	0.012	0.013	0.015	0.013	0.01	**0.113**
RA	0.048	0.068	0.015	0.022	0.149	0.012	0.018	0.046	0.012	0.016	0.015	0.014	0.011	**0.554**
PA	0.054	0.039	0.021	**0.595**	0.02	0.016	0.05	0.055	0.025	0.026	0.037	0.015	0.017	0.032
LE	0.029	0.022	0.023	0.018	0.018	0.015	0.017	0.027	**0.731**	0.02	0.023	0.028	0.014	0.017
HA	**0.349**	0.035	0.023	0.032	0.082	0.039	0.042	**0.163**	0.041	0.031	0.04	0.044	0.038	0.042

Distributions of allelic frequencies suggest that New York populations are distinct from Ontario populations, and that lake populations within the St. Lawrence drainage within New York are closely related to each other (histograms of allelic frequencies in Fig. S1). For all loci, the allele size range present in Ontario was present in New York. However, New York populations often contained an additional size range not found in Ontario. The allelic size range appeared bimodal for several loci: Lma29, Lmar18, RB7, and Lmar14. Geographic variation in allele size range was most dramatic for RB7. In this case, an allele size range of approximately 150–200 base pairs characterized Ontario populations, while an additional mode of alleles (250–300 base pairs) was common in New York populations.

### Shared and unique aspects of within-lake phenotypic divergence

**Figure 5:**
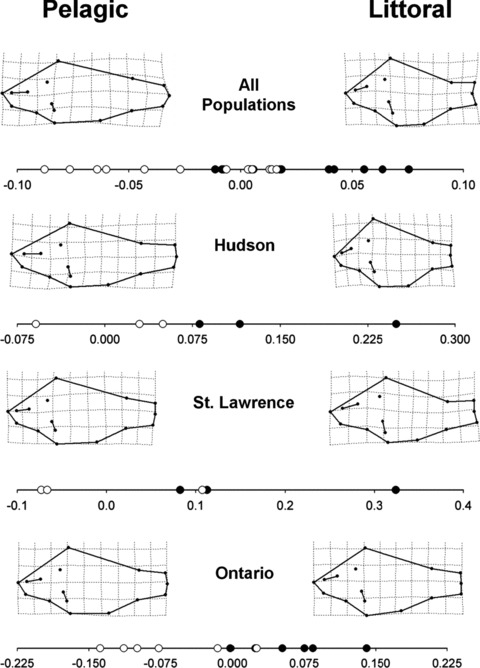
Shared features of body shape divergence between pelagic and littoral ecomorphs of pumpkinseed. Circles (filled = littoral, open = pelagic) indicate a particular population's mean canonical value extracted from the “ecomorph” canonical axis of the MANCOVA analyses (see Methods section). Thin-plate spline transformations represent the most extreme deviation from the consensus configuration (magnified ×2), and were generated by regressing canonical scores against shape data (*X*–*Y* coordinates) using TPSregr. As a result of finding significant interactions between ecomorph and genetic cluster in our analysis that included all populations (see Results), we also visualized divergence within each genetic cluster (St. Lawrence, Hudson, and Ontario).

The full MANCOVA analysis of all 12 lakes from the three genetic clusters detected a significant effect of ecomorph on morphological variation (Table 7), indicating that there is a shared aspect of divergence between littoral and pelagic habitats among all lakes. Pelagic ecomorphs had generally more fusiform bodies, narrower pectoral fin insertions, and perhaps larger eyes and mouth regions than littoral ecomorphs (Fig. 5). This result generally confirms the findings of earlier work in this system. However, our findings also demonstrate substantial additional variation in external body form in pumpkinseed sunfish that can be related to evolutionary history.

**Table 7:** MANCOVA analyses examining how shape variation is related to various factors. As a result of finding significant interactions between ecomorph and genetic cluster, separate MANCOVA analyses were performed for each genetic cluster (St. Lawrence, Hudson, and Ontario). Note that partial variance explained does not sum to unity (Langerhans and DeWitt 2004).

	F	df (numerator, denominator)	P	Wilk's partial λ	Partial variance explained (%)
All Populations					
Centroid size	41.33	28,445	0.0001	0.278	72.2
Ecomorph	4.53	28,445	< 0.0001	0.778	22.2
Genetic cluster	24.28	56,890	< 0.0001	0.156	60.4
Ecomorph × cluster	2.58	56,890	< 0.0001	0.740	13.9
Lake (Cluster)	7.45	252,3845	< 0.0001	0.033	26.7
St. Lawrence populations					
Centroid size	8.87	28,90	< 0.0001	0.266	73.4
Ecomorph	3.24	28,90	< 0.0001	0.498	50.2
Lake	7.50	56,180	< 0.0001	0.090	70.0
Ecomorph × lake	1.48	56,180	< 0.0283	0.469	31.5
Hudson populations					
Centroid size	8.11	28,89	< 0.0001	0.110	89.0
Ecomorph	2.17	28,89	0.0032	0.594	40.6
Lake	6.06	56,178	< 0.0001	0.118	65.6
Ecomorph × lake	1.18	56,178	0.214	0.533	27.0
Ontario populations					
Centroid size	13.89	28,201	< 0.0001	0.341	65.9
Ecomorph	6.68	28,201	< 0.0001	0.694	30.6
Lake	7.50	140,997	< 0.0001	0.029	83.0
Ecomorph × lake	3.79	140,997	< 0.0001	0.122	65.1

**Figure 6:**
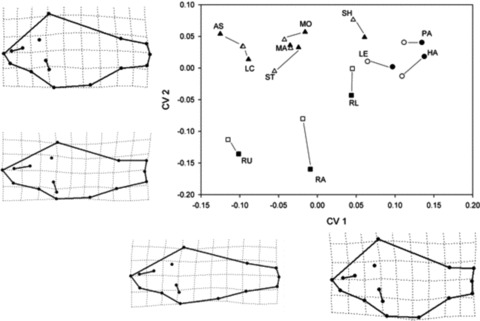
Features of body shape variation associated with genetic cluster membership (Ontario, St. Lawrence, and Hudson—see Results). Shapes represent different genetic clusters (filled = littoral ecomorphs, open = pelagic ecomorphs), triangles are Ontario populations, circles are Hudson populations, and squares are St. Lawrence populations. The *x* -axis represents mean body shape variation depicted by canonical variate 1 (CV1), the *y* -axis represents mean body shape variation depicted by canonical variate 2 (CV2), which were both extracted from the MANCOVA analysis that considered all populations (see Methods). Thin-plate spline transformations represent the most extreme deviation from the consensus configuration (magnified ×2) and were generated by regressing canonical scores against shape data (*X*–*Y* coordinates) using TPSregr.

In the full analysis of all 12 populations (Table 7) and apart from allometry (partial variance centroid size = 72.2%), the effect of genetic cluster accounted for the largest proportion of phenotypic variation (partial variance genetic cluster = 60.4%), a much higher proportion of phenotypic variation than was accounted for by the shared response to contemporary divergent selection between lake habitats (partial variance ecomorph = 22.2%), or by similarities among pumpkinseeds from the same lake (partial variance lake = 26.7%). In Figure 6 we visualize the canonical variates corresponding to the effect of genetic (historical) cluster. The first canonical variate (CV1) indicates that pumpkinseed from the Hudson genetic cluster have deeper bodies, wider pectoral insertion widths, and possibly smaller mouths compared to fish from the St. Lawrence and Ontario genetic clusters. Variation along CV2 indicates that pumpkinseed from the St. Lawrence cluster have narrower bodies, more ventrally placed pectoral fins, and larger mouths than pumpkinseeds from the Hudson and Ontario clusters. This is consistent with the significant interaction between ecomorph and cluster from the full MANCOVA model of all 12 populations that indicates unique aspects of morphological divergence among the three genetic clusters (Table 7). Therefore, in this system, patterns of morphological variation are influenced by both contemporary processes acting between divergent environments and evolutionary history.

Given this result, we were motivated to visualize axes of phenotypic divergence associated with habitat for populations in each genetic cluster, and so performed additional MANCOVA analyses only considering populations within each genetic cluster. In the St. Lawrence populations, variation in body depth was not associated with different lake habitats; but instead, divergence between pelagic and littoral habitats involved variation in the width and position of the pectoral fin insertions (Fig. 5). In contrast, among the Hudson and Ontario populations, the width and position of the pectoral fin insertion were similar for both ecomorphs (Fig. 5), but the body shape of the Littoral ecomorph was rounder and more gibbose—this result is more pronounced in the Hudson cluster compared to the Ontario cluster.

## Discussion

A central challenge in evolutionary biology is to understand how the interplay between historic and contemporary evolutionary process affects patterns of diversity within a species. Our population genetic analyses provide considerable insight into this interplay, because they revealed that while there is strong indirect evidence that diversifying selection acts on sympatric ecomorphs within lakes, contemporary lake populations of pumpkinseed also show obvious regional patterns of genetic structure. This raises the possibility that postglacial aspects of ancestry shapes phenotypic variation in two interesting ways. First, our morphometric analyses revealed strong associations between variation in body shape and genetic cluster membership. Second, unique aspects of within-lake phenotypic divergence occurred among genetic clusters, suggesting that diversification between lake habitats involves an interaction between postglacial history and contemporary natural selection within lakes. We discuss the evidence for various scales of historical divergence before focusing on how these have affected contemporary patterns of phenotypic diversity in pumpkinseed sunfish.

### Within- and among-lake patterns of population genetic structure

A major goal was to evaluate if contemporary populations of polyphenic pumpkinseed could have resulted from a single allopatric divergence between glacial refugia (e.g., Bernatchez 1997). We found no evidence of multiple colonizations following a single historical divergence, and instead our results are more consistent with within-lake divergence following colonization. The null hypothesis of allelic homogeneity between sympatric ecomorph populations was not rejected in nearly all lakes. Round and Lewey lakes showed some evidence of allelic heterogeneity, but multilocus F_st_ values between ecomorphs were very low (<0.008), and in only Round Lake was this different from zero (F_st_= 0.005). Expanded within-lake analyses of individual movement between habitats (McCairns and Fox 2004) may help us better understand the minimal neutral genetic divergence of sympatric ecomorphs (e.g., Fraser and Bernatchez 2005). Our individual-based clustering analyses using STRUCTURE also found little evidence of close relatedness among ecomorphs sharing the same lake habitat. In addition, we find private alleles shared between local ecomorph pairs in five populations (Harris, Rondaxe, Paradox, Round, and Lewey lakes). These data cause us to reject the ideas that ecologically and phenotypically divergent pumpkinseeds within lakes are distantly related and also reject that similar ecomorphs from different populations are closely related as expected under a single historical divergence hypothesis.

This conclusion is provisional, however, because we cannot reject all possible hypotheses regarding single evolutionary origins of ecomorphs within lakes without using a more powerful population genomic approach. For example, ecomorphs within lakes could arise from a single allopatric divergence followed by extensive within-lake introgression except at ecologically important genomic islands (Ellison et al. 2011), which, under persistent divergent selection, are not homogenized between ecomorphs. This seems less likely here though because we see little, if any, divergence in neutral alleles between ecomorphs in any lake populations despite a robust dataset involving strong replication within and among lakes. Moreover, diversifying selection arising between lake habitats is a key component of this hypothesis in order to maintain divergent phenotypes, and so even if this process had occurred, a role for diversifying selection on sunfish between lake habitats would still be supported.

While we found little evidence that contemporary pumpkinseed sunfish lake ecomorphs derive from a single historical divergence, we nonetheless found some evidence that ancestral pumpkinseed sunfish likely inhabited multiple glacial refugia. Complicated patterns of genetic structure and phylogeography are common for North American fish inhabiting formerly glaciated regions. Many fish colonized large geographical areas from various glacial refugia (Pielou 1991; Wilson and Hebert 1996; Bernatchez and Wilson 1998; Poissant et al. 2005; Aldenhoven et al. 2010). Specific dispersal outlets produced extensive zones of secondary contact between refugia lineages (Bernatchez and Dodson 1991; Bernatchez 1997; Wilson and Hebert 1996; Turgeon and Bernatchez 2001). After secondary contact, some reproductively isolated glacial lineages persisted in sympatry (Bernatchez 1997), while others resulted in extensive introgression (Turgeon and Bernatchez 2001; Lu et al. 2001). During the Wisconsian glaciation event, pumpkinseed sunfish were likely present in both the Mississippian and the Atlantic refugia (Underhill 1986; Mandrak and Crossman 1992) and so may have colonized central Ontario and New York state from either or both (Smith 1985; Underhill 1986; Mandrak and Crossman 1992). Our analyses suggest that Ontario populations of pumpkinseed received a greater genetic contribution from Mississippian ancestors, while New York populations received a greater contribution from Atlantic refugial ancestors, although introgression between lineages likely occurred in both regions. Pumpkinseed populations in New York contain an additional allele size range not present in Ontario populations that was also bimodal for four loci in the New York populations (Fig. S1).

Our postglacial dispersal hypothesis of pumpkinseed sunfish is similar to one proposed for lake trout in eastern North America. Wilson and Hebert (1996) found that Atlantic haplotypes dominated in New York populations while Mississippian haplotypes dominated central Ontario populations of lake trout. While generally classified as a warm water fish, pumpkinseed sunfish are tolerant of cooler waters, having the lowest critical thermal maxima of *Lepomid* taxa (Beitinger et al. 2000, Coker et al. 2001), and so perhaps dispersed somewhat like the cold-tolerant lake trout across the postglacial landscape. A more genomically and geographically extensive sampling effort ideally incorporating a more nuanced understanding of periglacial waterways (i.e., Poissant et al. 2005), is required to further evaluate the geographical origins and dispersal of pumpkinseed sunfish ancestors in the region.

A significant insight of this study is the finding that population genetic structure reflected geographical entities. Our AMOVA analyses indicated that both lakes and drainages explained significant allelic variation. All pairwise estimates of F_st_ and R_st_ between lakes were significantly greater than zero (F_st_ range = 0.025–0.198; R_st_ range 0.016–0.792). Also, the STRUCTURE analysis identified *K*= 14 as the most likely number of clusters (two more clusters than lakes; although clusters were generally associated with particular lakes). Yet, we found no evidence that the magnitude of genetic differentiation among lake populations was related to distance between lakes along contemporary watersheds (e.g., Crispo and Chapman 2008). This conflict largely arises from the tendency of Ontario populations in the Trent and Ottawa drainages to be closely related, despite the relative proximity of the Trent drainage to the St. Lawrence drainage in New York (Fig. 1). The young geological ages of these contemporary waterways may not have provided enough time to develop IBD patterns (Bernatchez and Wilson 1998).

Our population genetic analyses also persistently found that lake populations clustered into three larger geographical entities: Ontario, the south shore of the St. Lawrence River, and the Hudson River drainages. The neighbor-joining phenogram based on D_ce_ distances grouped lake populations to these three geographical entities and this was corroborated by a series of constrained STRUCTURE models (Garnier et al. 2004; Crispo and Chapman 2008; Aldenhoven et al. 2010). A model constrained to *K*= 2 separated sunfish between Ontario and New York, while a *K*= 3 model distinguished sunfish from the regions of Ontario, St. Lawrence, and the Hudson River drainages. The absence of detailed evidence for IBD along contemporary waterways above, however, suggests that our study lakes were colonized along different historical rather than contemporary waterways (Poissant et al. 2005), such as mobile proglacial lakes that followed the retreating edge of the glaciers (e.g., Mandrak and Crossman 1992). While this regional population genetic structure could result from neutral and/or unknown adaptive evolutionary processes, this geographic pattern is nonetheless well supported and provides an opportunity to evaluate its effects on contemporary phenotypic diversification of pumpkinseed sunfish.

### Contemporary and historical influences on phenotypic variation

Previous studies documenting parallel aspects of phenotypic divergence among lakes containing polyphenic populations of pumpkinseed (Robinson et al. 2000; Jastrebski and Robinson 2004; Gillespie and Fox 2003; Riopel et al. 2008) have assumed that lake populations are evolutionarily independent and that historical effects have little affect on phenotypic diversity. Our analyses above show that neither of these assumptions is strictly justified because all populations are related, although in a nested hierarchy that affords less evolutionary independence between lakes within regional genetic clusters and greater independence among lakes from different genetic clusters. Therefore, does incorporating our understanding of these historical effects help us better understand the nature of polyphenism in pumpkinseed sunfish? We evaluated the influence of history on body shape variation in this system by: (1) comparing the phenotypes of pumpkinseeds from different genetic clusters (identified by our analyses of microsatellite allele frequencies), and (2) testing for unique and shared aspects of within-lake divergence among these genetic clusters (Langerhans and DeWitt 2004; Hendry et al. 2006; Ward and Mclennan 2009).

We continue to find evidence of parallel diversification in some aspects of body form between lake habitats after accounting for history (a significant effect of ecomorph) that is consistent with earlier work (Robinson et al. 2000; Jastrebski and Robinson 2004). Pelagic ecomorphs generally have more streamlined bodies and narrower insertions of the pectoral fins compared to littoral ecomorphs (Fig. 5). These differences are consistent with established form–function relationships between fish body shape and swimming performance (reviewed in Jastrebski and Robinson 2004; Riopel et al. 2008). A more streamlined body may permit more efficient locomotion, but also reduces maneuverability (Webb 1984; Langerhans et al. 2003). Thus, pelagic pumpkinseeds that forage on aggregates of zooplankton dispersed in the large but structurally less complicated pelagic habitat may be well served by a body shape emphasizing swimming efficiency at the expense of maneuverability. Pectoral fins are used by sunfish for turning and braking and also contribute importantly to paired fin locomotion (Drucker and Lauder 2001, 2002), especially when precise movements are required in structurally complex habitats (but see Higham et al. 2005). Thus, littoral pumpkinseeds may be well served by wider pectoral fins if they reflect an increased reliance upon precise, pectoral fin locomotion (Ehlinger and Wilson 1988).

Our results suggest that the littoral–pelagic polyphenism is replicated because lineages of pumpkinseed sunfish become increasingly evolutionarily independent especially between biogeographic regions, while polyphenism is still found in many lakes. Replicated phenotypic polyphenisms support the role of divergent selection between littoral and pelagic habitats in many of the postglacial lakes of central Ontario and the Adirondack region of New York State. However, there was little evidence of heterogeneity in allelic frequency between ecomorphs in most lake populations, indicating that sympatric ecomorphs have not been sufficiently reproductively isolated for neutral alleles to become ecologically isolated at this scale as in other studies (Crispo and Chapman 2008). While we have found evidence that ancestral populations of pumpkinseed sunfish likely diverged between glacial refugia and subsequently colonized postglacial lakes, contemporary processes occurring within lakes continue to shape the phenotypic diversity between coexisting sunfish ecomorphs.

Despite numerous studies that have documented associations between neutral genetic variation and phenotypic variation (Merilä 1997; Hendry et al. 2002; Nicholls et al. 2006; Ward and Mclennan 2009), we were nonetheless surprised that the effect of postglacial colonization history (genetic cluster) also seems to influence body shape, and do so more strongly than either the effect of lake habitat (ecomorph) or lake population (full analysis of all 12 lake populations after accounting for allometry; Table 7). We visualized the body shape differences associated with genetic cluster membership, and found that the first two canonical variates both emphasized variation in body depth. Pumpkinseeds from the Hudson River cluster tend to have the greatest body depths, while pumpkinseed from Ontario had deeper bodies than those from the St. Lawrence genetic cluster. Evolutionary history and contemporary patters of selection may be confounded such that the geographic entities associated with our genetic clusters (Ontario, Hudson river drainage, St. Lawrence river drainage) differ in contemporary environmental features. This limits our ability to conclude that differences in morphology associated with genetic cluster are exclusively caused by historical evolutionary processes. However, we know of no obvious contemporary ecological difference (i.e., in aquatic community structure or physical habitat characteristics) of lakes among these regions or drainages that could produce these effects (latitudinal differences between Ontario and New York may also be offset by higher altitudes of lakes in the Adirondack region of New York relative to Ontario). A comprehensive limnological survey of a subset of lakes representing each geographical region could usefully address this issue. Our preliminary conclusion is that it seems more reasonable that regional genetic clusters reflects ancestral lineages that diverged under selection, genetic drift, or founder events either between glacial refugia or during the dispersal phase that preceded the colonization of contemporary lakes (Ward and Mclennan 2009).

Evolutionary history has also influenced the contemporary process of phenotypic divergence between littoral and pelagic lake habitats (an interaction between genetic cluster and ecomorph; Table 7). To better understand patterns of phenotypic divergence in each regional cluster, we visualized the canonical variates associated with ecomorph from three cluster-specific MANCOVAs. Comparisons revealed some interesting insights regarding interactions between postglacial history and responses to contemporary selective regimes between lake habitats. Within the St. Lawrence cluster, differences between pelagic and littoral pumpkinseeds stress variation in the width of the pectoral fin insertion (consistent with the results of the analysis that included all populations, St. Lawrence littoral ecomorphs had wider pectoral fin insertions). However, in contrast to our all-population analysis, the St. Lawrence littoral ecomorphs also seem to be more streamlined than their local pelagic counterparts. Within the Hudson and Ontario clusters, the pectoral fin insertion width seems to be relatively invariant between ecomorphs, but pelagic ecomorphs have more streamlined body shapes than their littoral counterparts. Thus, depending on their postglacial evolutionary history, pumpkinseeds can evolve diverse morphological solutions to divergent selection between littoral and pelagic habitats. Improvements to maneuverability (favored in the littoral habitat) can perhaps be made by modifying the width of pectoral fin insertions and/or by increasing body depth. Conclusions about the exclusive effects of history are again provisional because some cryptic contemporary ecological differences between these geographical entities may be confounded with historical evolutionary effects. Assessing contemporary patterns of selection in replicated lake populations nested within the different genetic clusters would address this hypothesis. Selection data (the relationship between traits and fitness), could be combined into a single selection analysis (Weese et al. 2010) in order to test for similarities in the pattern and strength of selection among lakes nested within genetic clusters.

It is important to acknowledge that since our morphological analyses involved wild caught fish, we cannot discriminate between genetic versus environmental variation, although this has been the focus of substantial previous research in this system. Rearing studies demonstrate that a combination of minor genetic and substantial plastic developmental responses to dietary and predator cues shapes pumpkinseed sunfish phenotype including between these ecomorphs (Robinson and Wilson 1996). Of greater interest, is the finding that the plastic developmental responses themselves have diverged between sympatric ecomorphs (Parsons and Robinson 2006, 2007; Januszkiewicz and Robinson 2007; Robinson et al. 2008). The near absence of genetic differentiation between sympatric ecomorphs at neutral loci here combined with heritable differences between sympatric ecomorphs raises the possibility that certain functional loci may adaptively diverge between ecomorphs perhaps via a “porous genome” phenomenon (Barton and Bengtsson 1986, Wu 2001). Under this model, early in a divergence, only functional loci under diversifying selection diverge while neutral loci remain homogenized for much longer between sympatric populations (Via and West 2008). If occurring here, then it is interesting that the functional loci apparently diverging between sympatric pumpkinseed ecomorphs influence plastic morphological responses to littoral versus pelagic conditions. The role of phenotypic plasticity in adaptive divergence is incompletely understood (Ghalambor et al 2007; Pfennig et al 2010; Moczek et al 2011), and it can either enhance or constrain adaptive divergence through its effect on gene flow among subpopulations experiencing diversifying selection (Price et al 2003). Evaluating the porous genome hypothesis and the role of plastic traits here will require identifying nonneutral genetic markers under diversifying selection between lake habitats and then testing their relationship to the divergence of plastic morphological responses between ecomorphs.

Our study documents how the variable evolutionary histories of different populations diverging along a similar environmental gradient produce both shared and unique aspects of divergence. In addition, our understanding of these new regional effects on phenotype within pumpkinseed sunfish fills a gap in an array of historical effects that range from deep effects identified among Lepomid taxa predating that last glacial period (Riopel et al. 2008), more recent historic glacial effects on meristic characters between eastern and western populations of pumpkinseed sunfish over their range (Scott and Crossman 1998), and the contemporary processes of diversification occurring within and among lakes (Robinson et al. 2000; Gillespie and Fox 2004; Jastrebski and Robinson 2004). By incorporating population genetic with morphometric analytic methods, we have highlighted how contemporary patterns of phenotypic divergence are better understood against a known backdrop of evolutionary history.

## Supporting Information

**Table S1.** Summary of genetic polymorphism detected at six microsatellite loci from 24 ecotype populations of pumpkinseed sunfish in central Ontario and the Adirondack region of New York (for population abbreviations refer to Figure 1).

**Figure S1.** Allele frequencies of six microsatellite loci in the three major genetic clusters (see Results) that contain the 12 lakes considered in this study.
