# Supplementary material for: Contemporary and historical evolutionary processes interact to shape patterns of within-lake phenotypic divergences in polyphenic pumpkinseed sunfish, Lepomis gibbosus
Source: Ecol Evol. 2012 Mar;2(3):574–92. doi: 10.1002/ece3.72 (PMC3399146; doi:10.1002/ece3.72)

Table S1 - Summary of genetic polymorphism detected at 6 microsatellite loci from 24 ecotype populations of pumpkinseed sunfish in Central Ontario and the Adirondack region of New York (for population abbreviations refer to Figure 1). Littoral populations are indicated by (/l), pelagic populations are indicated by (/p). Parameters estimated are: Number of individuals successfully genotyped (N), number of alleles (A), expected heterozygosity (H_e_), and observed heterozygosity (H_o_) are given. H_o_ values that significantly differ from Hardy-Weinberg expectations are indicated by * (*P* <0.05).

|  |  | Locus |  |  |  |  |  |
| --- | --- | --- | --- | --- | --- | --- | --- |
| Population | Parameters | Lma29 | RB20 | Lmar9 | Lmar18 | RB7 | Lmar14 |
|  | N | 46 | 46 | 44 | 44 | 48 | 43 |
| AS/l |
|  | A | 3 | 11 | 7 | 8 | 16 | 14 |
|  | H_e_ | 0.16 | 0.87 | 0.55 | 0.86 | 0.89 | 0.86 |
|  | H_o_ | 0.17 | 0.93* | 0.57 | 0.89 | 0.92 | 0.40* |
|  |  |  |  |  |  |  |  |
| AS/p | N | 47 | 47 | 45 | 47 | 48 | 41 |
|  | A | 3 | 11 | 6 | 8 | 15 | 13 |
|  | H_e_ | 0.12 | 0.87 | 0.51 | 0.85 | 0.9 | 0.9 |
|  | H_o_ | 0.13 | 0.81 | 0.47 | 0.91 | 0.92 | 0.51* |
|  |  |  |  |  |  |  |  |
| MA/l | N | 47 | 46 | 48 | 45 | 47 | 38 |
|  | A | 3 | 11 | 6 | 10 | 17 | 5 |
|  | H_e_ | 0.08 | 0.87 | 0.8 | 0.81 | 0.89 | 0.59 |
|  | H_o_ | 0.09 | 0.91 | 0.79 | 0.87 | 0.94 | 0.24* |
|  |  |  |  |  |  |  |  |
| MA/p | N | 47 | 46 | 48 | 44 | 47 | 37 |
|  | A | 2 | 13 | 7 | 9 | 15 | 7 |
|  | H_e_ | 0.06 | 0.88 | 0.8 | 0.83 | 0.88 | 0.69 |
|  | H_o_ | 0.06 | 1 | 0.77 | 0.82 | 0.91 | 0.14* |
|  |  |  |  |  |  |  |  |
| ST/l | N | 48 | 48 | 48 | 48 | 48 | 48 |
|  | A | 2 | 8 | 4 | 7 | 12 | 14 |
|  | H_e_ | 0.4 | 0.71 | 0.66 | 0.72 | 0.76 | 0.86 |
|  | H_o_ | 0.25* | 0.65 | 0.63 | 0.79 | 0.79 | 0.88 |
|  |  |  |  |  |  |  |  |
| ST/p | N | 48 | 48 | 47 | 47 | 48 | 45 |
|  | A | 2 | 8 | 6 | 6 | 14 | 10 |
|  | H_e_ | 0.43 | 0.69 | 0.61 | 0.72 | 0.77 | 0.83 |
|  | H_o_ | 0.48 | 0.75 | 0.49 | 0.72 | 0.73 | 0.93 |
|  |  |  |  |  |  |  |  |
| MO/l | N | 34 | 34 | 34 | 32 | 34 | 32 |
|  | A | 2 | 6 | 5 | 7 | 8 | 7 |
|  | H_e_ | 0.4 | 0.67 | 0.65 | 0.7 | 0.7 | 0.58 |
|  | H_o_ | 0.41 | 0.79 | 0.62 | 0.63 | 0.74 | 0.34* |
|  |  |  |  |  |  |  |  |
| MO/p | N | 48 | 48 | 45 | 47 | 47 | 41 |
|  | A | 2 | 5 | 4 | 6 | 9 | 7 |
|  | H_e_ | 0.43 | 0.64 | 0.6 | 0.71 | 0.67 | 0.72 |
|  | H_o_ | 0.42 | 0.73 | 0.67 | 0.72 | 0.77 | 0.39* |
|  |  |  |  |  |  |  |  |
| LC/l | N | 47 | 47 | 48 | 48 | 47 | 44 |
|  | A | 2 | 12 | 11 | 10 | 15 | 10 |
|  | H_e_ | 0.51 | 0.84 | 0.86 | 0.81 | 0.89 | 0.81 |
|  | H_o_ | 0.51 | 0.89 | 0.9 | 0.9 | 0.94 | 0.55* |
|  |  |  |  |  |  |  |  |
| LC/p | N | 48 | 42 | 48 | 38 | 46 | 44 |
|  | A | 3 | 13 | 11 | 8 | 17 | 14 |
|  | H_e_ | 0.51 | 0.87 | 0.89 | 0.79 | 0.88 | 0.9 |
|  | H_o_ | 0.58 | 0.98 | 0.79 | 0.89 | 0.93 | 0.82* |
|  |  |  |  |  |  |  |  |
| SH/l | N | 47 | 40 | 47 | 44 | 46 | 40 |
|  | A | 3 | 16 | 9 | 12 | 17 | 16 |
|  | H_e_ | 0.16 | 0.9 | 0.85 | 0.89 | 0.92 | 0.93 |
|  | H_o_ | 0.17 | 0.98 | 0.89* | 0.93 | 0.89 | 0.28* |
|  |  |  |  |  |  |  |  |
| SH/p | N | 47 | 47 | 47 | 45 | 47 | 30 |
|  | A | 2 | 13 | 10 | 13 | 16 | 17 |
|  | H_e_ | 0.12 | 0.88 | 0.84 | 0.9 | 0.92 | 0.93 |
|  | H_o_ | 0.13 | 0.94 | 0.83 | 0.96 | 0.98 | 0.37* |
|  |  |  |  |  |  |  |  |
| RL/l | N | 45 | 46 | 42 | 46 | 45 | 43 |
|  | A | 6 | 14 | 10 | 17 | 27 | 14 |
|  | H_e_ | 0.55 | 0.91 | 0.78 | 0.87 | 0.94 | 0.88 |
|  | H_o_ | 0.53 | 0.89 | 0.9 | 0.89 | 0.93 | 0.79 |
|  |  |  |  |  |  |  |  |
| RL/p | N | 46 | 44 | 45 | 46 | 46 | 45 |
|  | A | 5 | 15 | 9 | 15 | 24 | 17 |
|  | H_e_ | 0.54 | 0.91 | 0.78 | 0.89 | 0.94 | 0.93 |
|  | H_o_ | 0.39 | 0.91 | 0.82 | 0.98 | 0.85* | 0.89* |
|  |  |  |  |  |  |  |  |
| RU/l | N | 47 | 44 | 46 | 34 | 42 | 38 |
|  | A | 4 | 14 | 11 | 20 | 21 | 15 |
|  | H_e_ | 0.52 | 0.91 | 0.83 | 0.94 | 0.93 | 0.9 |
|  | H_o_ | 0.51 | 0.89 | 0.89 | 1 | 0.9 | 0.87 |
|  |  |  |  |  |  |  |  |
| RU/p | N | 47 | 46 | 48 | 48 | 46 | 44 |
|  | A | 2 | 14 | 12 | 23 | 25 | 14 |
|  | H_e_ | 0.48 | 0.91 | 0.78 | 0.95 | 0.94 | 0.92 |
|  | H_o_ | 0.51 | 0.8 | 0.77 | 0.98 | 0.91 | 0.89 |
|  |  |  |  |  |  |  |  |
| RA/l | N | 45 | 48 | 47 | 48 | 48 | 48 |
|  | A | 5 | 17 | 11 | 18 | 24 | 16 |
|  | H_e_ | 0.5 | 0.88 | 0.86 | 0.87 | 0.92 | 0.89 |
|  | H_o_ | 0.47 | 0.9 | 0.81 | 0.92 | 0.88 | 0.71* |
|  |  |  |  |  |  |  |  |
| RA/p | N | 45 | 46 | 45 | 42 | 46 | 46 |
|  | A | 4 | 19 | 11 | 17 | 27 | 15 |
|  | H_e_ | 0.36 | 0.91 | 0.85 | 0.85 | 0.92 | 0.91 |
|  | H_o_ | 0.38 | 0.89 | 0.93 | 0.88 | 0.91 | 0.70* |
|  |  |  |  |  |  |  |  |
| PA/l | N | 46 | 47 | 47 | 46 | 46 | 41 |
|  | A | 6 | 11 | 10 | 15 | 22 | 18 |
|  | H_e_ | 0.76 | 0.87 | 0.82 | 0.9 | 0.91 | 0.93 |
|  | H_o_ | 0.8 | 0.89 | 0.91 | 0.93 | 0.91 | 0.9 |
|  |  |  |  |  |  |  |  |
| PA/p | N | 44 | 44 | 44 | 46 | 48 | 47 |
|  | A | 6 | 15 | 9 | 14 | 17 | 21 |
|  | H_e_ | 0.72 | 0.88 | 0.84 | 0.86 | 0.91 | 0.91 |
|  | H_o_ | 0.64 | 0.98 | 0.86 | 0.93 | 0.94 | 0.79 |
|  |  |  |  |  |  |  |  |
| LE/l | N | 47 | 47 | 46 | 44 | 48 | 41 |
|  | A | 2 | 13 | 9 | 13 | 12 | 18 |
|  | H_e_ | 0.46 | 0.79 | 0.72 | 0.88 | 0.79 | 0.92 |
|  | H_o_ | 0.45 | 0.87 | 0.63* | 0.91* | 0.85 | 0.93 |
|  |  |  |  |  |  |  |  |
| LE/p | N | 48 | 47 | 46 | 46 | 48 | 43 |
|  | A | 3 | 14 | 8 | 13 | 14 | 17 |
|  | H_e_ | 0.51 | 0.85 | 0.77 | 0.85 | 0.72 | 0.92 |
|  | H_o_ | 0.42 | 0.96 | 0.74 | 0.85 | 0.67 | 0.93 |
|  |  |  |  |  |  |  |  |
| HA/l | N | 47 | 45 | 46 | 45 | 44 | 47 |
|  | A | 6 | 12 | 9 | 13 | 25 | 19 |
|  | H_e_ | 0.64 | 0.9 | 0.81 | 0.86 | 0.94 | 0.93 |
|  | H_o_ | 0.57* | 1 | 0.91 | 0.93 | 0.93 | 0.94 |
|  |  |  |  |  |  |  |  |
| HA/p | N | 45 | 45 | 45 | 44 | 45 | 45 |
|  | A | 6 | 11 | 9 | 18 | 28 | 22 |
|  | H_e_ | 0.56 | 0.85 | 0.84 | 0.9 | 0.95 | 0.94 |
|  | H_o_ | 0.6 | 0.8 | 0.69* | 0.89 | 1 | 0.89 |

Figure S1. Allele frequencies of 6 microsatellite loci in the 3 major genetic clusters (see Results) that contain the 12 lakes considered in this study. Allele frequency (y-axis) and allele size in base pairs (x-axis) are given for each locus.


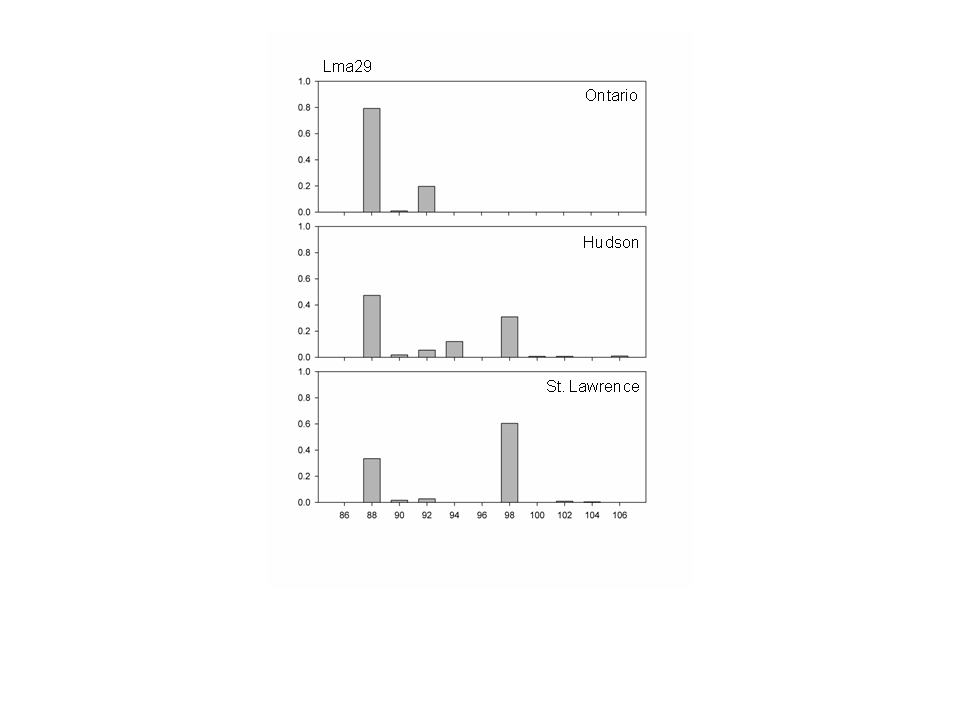


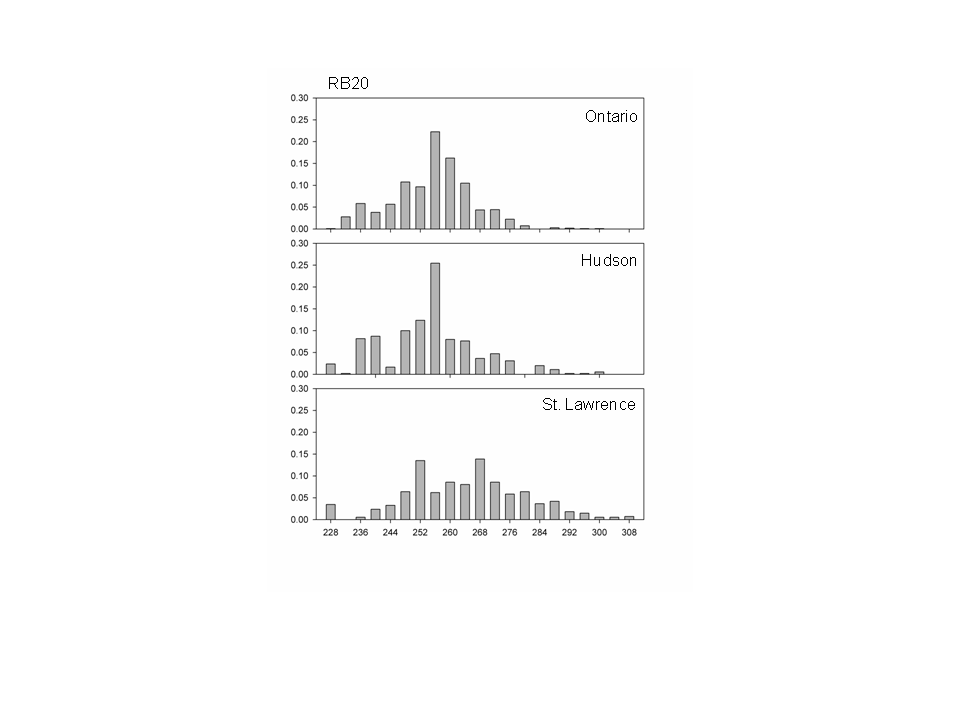


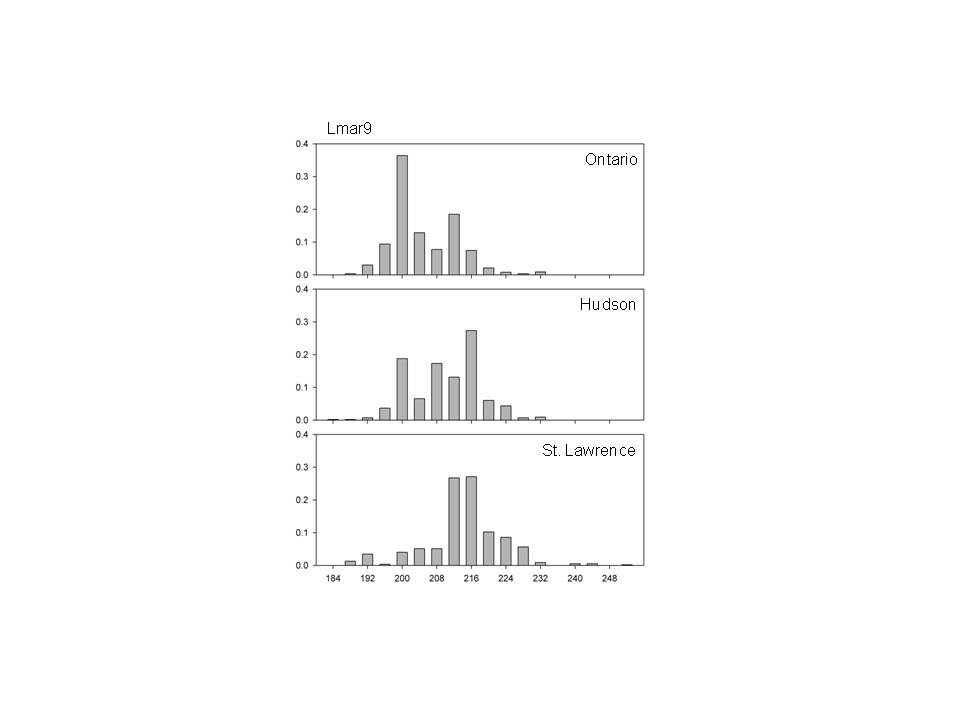


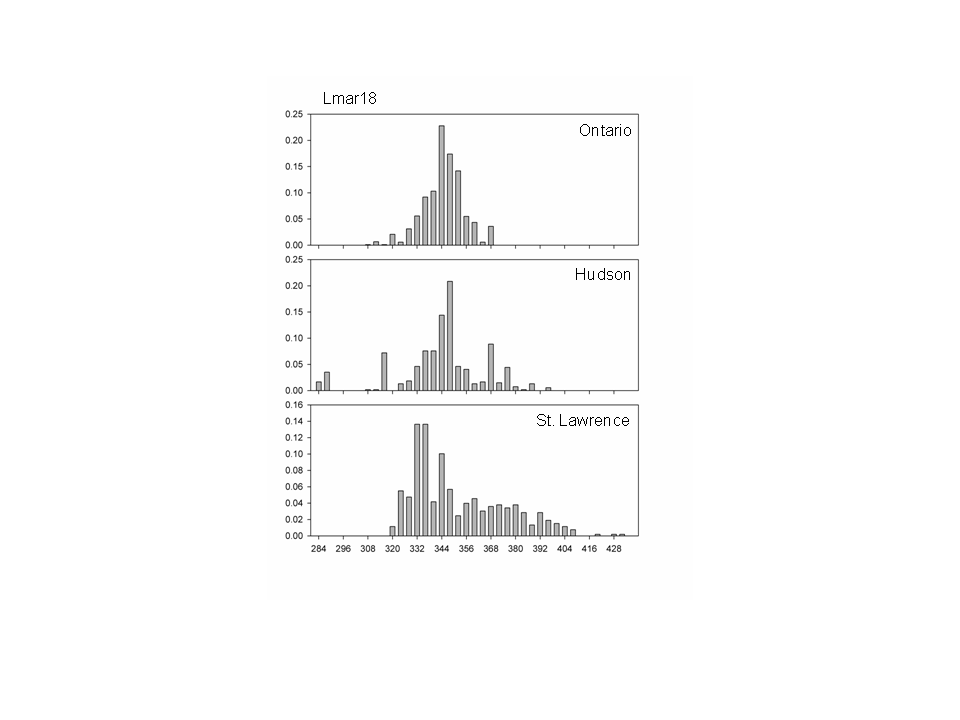


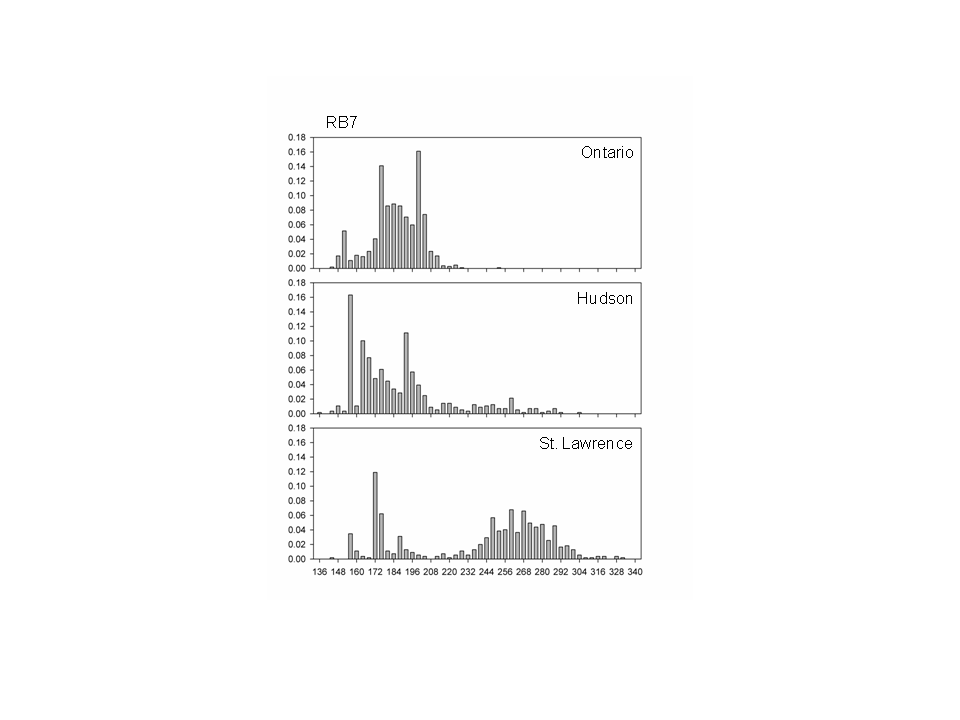


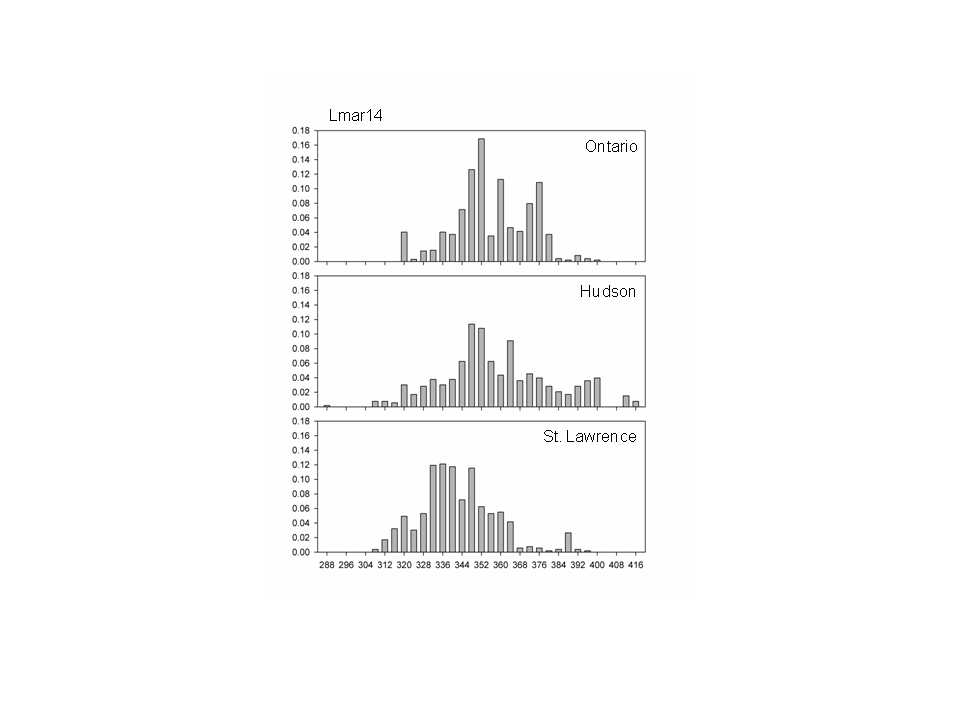

Supplement: Supplementary file 1 [file ece30002-0574-SD1.doc]
